# Post-Blunt Traumatic Hemobilia From Pseudoaneurysm Successfully Treated With Embolization

**DOI:** 10.7759/cureus.7961

**Published:** 2020-05-05

**Authors:** Pham Hong Duc, Pham Xuan Dung, Huynh Quang Huy

**Affiliations:** 1 Radiology, Ha Noi Medical University, Ha Noi, VNM; 2 Oncology, Ho Chi Minh City Oncology Hospital, Ho Chi Minh, VNM; 3 Radiology, Pham Ngoc Thach University of Medicine, Ho Chi Minh, VNM

**Keywords:** hemobilia, blunt liver trauma, pseudoaneurysm, embolisation

## Abstract

Hemobilia is an uncommon but serious complication of traumatic liver injury. Diagnosis can be difficult because the presentation of gastrointestinal (GI) bleeding may be minimal or massive, and its delay is quite variable, ranging from a few days to several months. Radiologic evaluation, including ultrasound, CT, endoscopic retrograde cholangiopancreatography, and angiography, can be used for the diagnosis. Once the clinical presentation and diagnosis are considered, diagnostic and therapeutic arteriography must be performed as soon as possible. Here, we present a case of hemobilia two weeks postoperative for blunt traumatic liver injury in a 13-year-old boy who was treated successfully by N-butyl 2-cyanoacrylate (NBCA) embolization of the pseudoaneurysm.

## Introduction

The liver is one of the most frequently injured organs in blunt abdominal trauma. Hemobilia is a rare complication following abdominal trauma; only 6% of cases had trauma (of which blunt trauma is 5%), while the most common is iatrogenic (65%) such as percutaneous liver procedures or biopsies, other causes (29%) include biliary lithiasis, inflammation, vascular malformations, parasitic infections, and tumors [1]. Recently, Guana et al., in a series of 116 young patients who sustained blunt trauma with liver injury, found only one case of hemobilia (0.9%) [2]. Delayed complications such as hemobilia, bilomas, bile leaks, or abscesses are uncommon [3]. Among them, hemobilia is an extremely rare complication, and only isolated cases have been reported in the literature. Depending on its suspected source as well as severity, the treatment of hemobilia could range from medical therapy to endoscopy, interventional radiology, or surgery. Transarterial hepatic intervention is safe and effective to stop the bleeding whenever it is severe or recurrent. Here, we present a case of hemobilia two weeks postoperative for blunt traumatic liver injury in a 13-year-old boy who was treated successfully by N-butyl 2-cyanoacrylate (NBCA) embolization of the pseudoaneurysm and a review of the literature.

## Case presentation

A 13-year-old boy presented with an abdominal injury after suffering a bicycle accident. He underwent open surgery for hemoperitoneum of hepatic laceration at a provincial hospital. The child stabilized hemodynamically two weeks after surgery. Before discharge, the child experienced severe vomiting of blood and passage of black tarry stools. Inspite of multiple blood transfusions, his anemia did not improve until after one week of resuscitation. The patient was referred to our hospital. During four days of hospitalization, he had continuous abdominal pain and hematemesis. Though the patient received three units (750 mL) of blood, laboratory studies showed anemia, red blood cells of 2.82 x 109/L, hemoglobin of 71 g/L, and hematocrit of 22%. No sign of cholestatic jaundice was found with a total bilirubin of 20 µmol/L (direct bilirubin was 15.6 µmol/L). Aminotransferase was mildly elevated with serum glutamic-oxaloacetic transaminase of 301 IU/L and serum glutamic pyruvic transaminase of 199 IU/L. On examination, the child was pale but afebrile, no clear icterus, and had a mildly tender abdomen in the right upper quadrant. Radiographic methods have been indicated.

**Figure 1:**
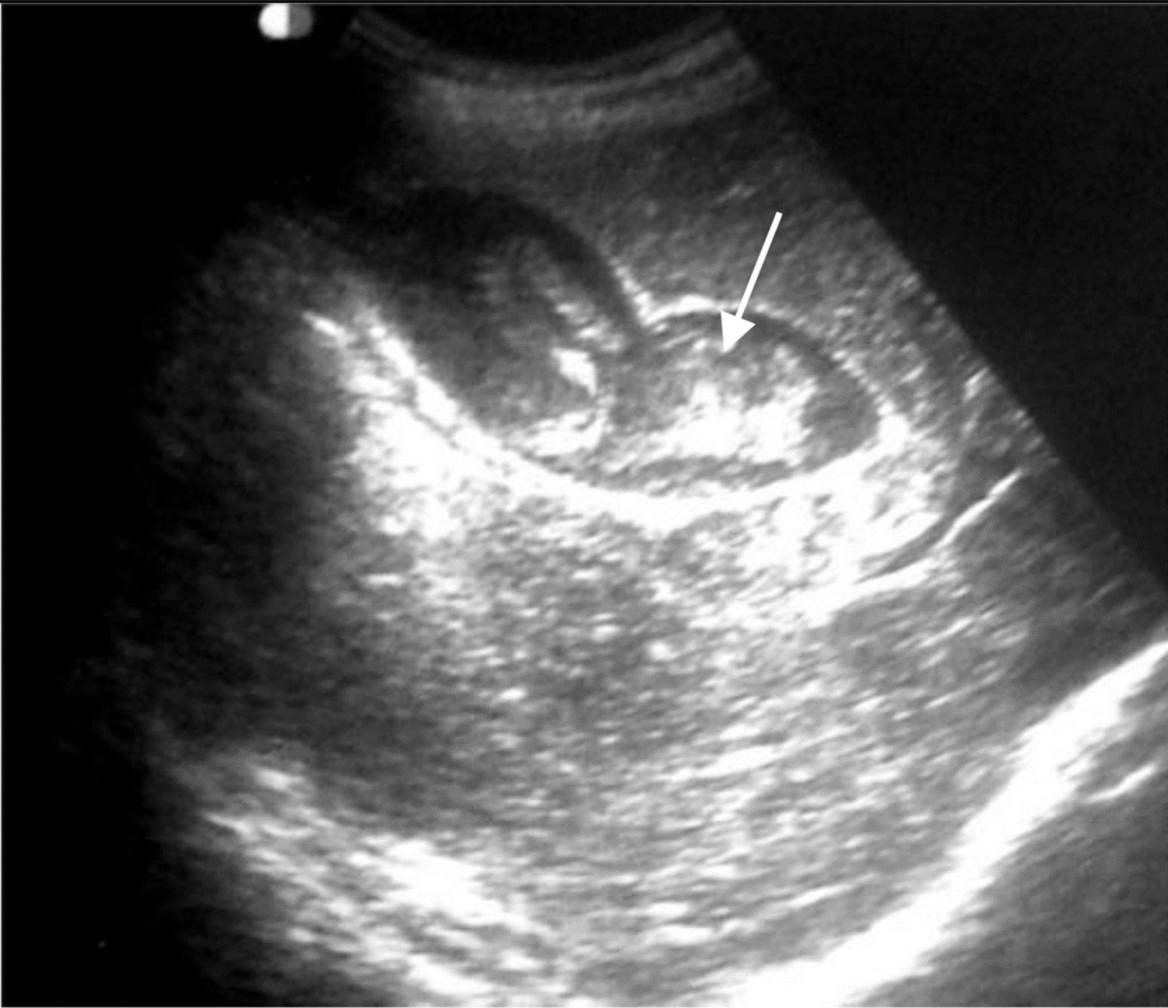
Ultrasound examination. The gallbladder showed an echogenic blood clot (arrow) in the lumen.

**Figure 2:**
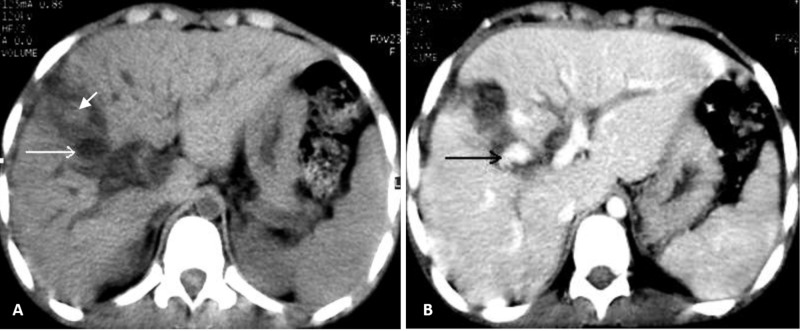
Hepatic axial CT scan. This hepatic axial CT scan without (A) and with (B) contrast showed a hypodense defect in the anterior segment of the right lobe with a slight spontaneously hyperdense blood clot (A, short arrow). The presence of a well-defined hypodense (A, long arrow) and enhanced formation (B, long arrow) suggested a pseudoaneurysm. Note the slightly dilated intrahepatic bile ducts.

Abdominal ultrasound revealed a heterogenous septate fluid collection within the parenchyma of right lobe presumed to be due to old hematomas from laceration of liver, subtle dilation of intrahepatic and extrahepatic bile ducts, and a distended gallbladder with an echogenic blood clot in the lumen (Figure 1). Unfortunately, color Doppler had not been performed on this patient to confirm a false intrahepatic aneurysm. We noted no collection or fluid of perihepatic and intraabdominal cavities. An abdominal CT scan without and with contrast medium demonstrated a hepatic cavity in the right lobe of the liver with the density of regressive hematoma. Deep within this old laceration and near the right branch of the portal vein showed an enhanced lesion, strongly suggesting a pseudoaneurysm of the right branch of the hepatic artery (Figure 2).

**Figure 3:**
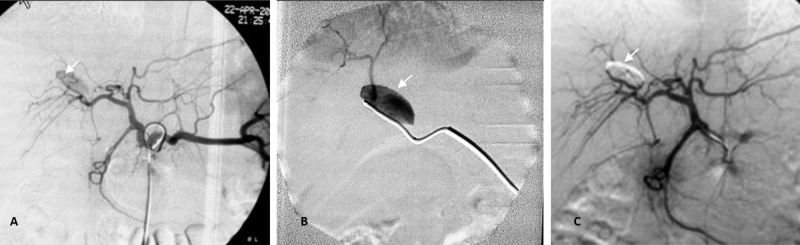
Arteriography with embolization. This is a celiac trunk angiography identifying a pseudoaneurysm of anterior segment branch of the right hepatic artery (A), confirmed by superselective feeder (B). Selective arteriography after embolization showed complete occlusion of the pseudoaneurysm sac by NBCA mixed with lipiodol (1:3 ratio) (C). NBCA, N-butyl 2-cyanoacrylate

The next day, the interventional radiology team performed a hepatic angiogram under general anesthesia. The hepatic arteriogram revealed a pseudoaneurysm of the anterior segment branch of the right hepatic artery with no active extravasation. A 2.4-F microcatheter was introduced coaxially into the 5-F catheter, and the tip of the microcatheter was placed just to the neck of the pseudoaneurysm. Selective angiograms by manual injection of several milliliters of contrast with very slight pressure to avoid ruptured pseudoaneurysm clearly showed an aneurysm with its downstream arteries. A histoacryl (NBCA) and lipiodol mixture at a ratio of 1:3 (25% NBCA) was injected under careful fluoroscopic monitoring until it filled the outflow. After then, the microcatheter was immediately removed to prevent adherence of the microcatheter tip to the artery wall. Post-embolic angiography showed complete occlusion of the pseudoaneurysm, no retrograde reflux of parent artery inflow; a few drops of glue migrated into downstream arteries but were not significant (Figure 3).

Daily laboratory values showed a gradual improvement of his anemia and his liver function tests. He was discharged seven days after the procedure. At follow-up one month later, the patient was recovering without further complications proven by ultrasound and CT.

## Discussion

The mechanism of hemobilia due to a ruptured liver is due to an abnormal communication between an intrahepatic blood vessel and the biliary system developing into a venobiliary or arteriobiliary fistula. Arteriobiliary fistulae are a very common cause of hemobilia, as a result of vessel wall damage, with subsequent necrosis and rupture into the biliary ducts [1, 3]. Thus, hemobilia often occurs in liver laceration of a high-grade III-IV. However, one report also showed a rare case of hemobilia but without any liver parenchymal injury on CT scan [4].

The time to the presentation of hemobilia after the initial trauma is also quite variable relative to the initial injury as it may be delayed for days to several months; we found a range from two days to 120 days in the literature [5-6]. Clinical manifestations of hemobilia were classically the triad of Quincke, including gastrointestinal (GI) bleeding, abdominal pain, and jaundice, but is rarely seen in all cases [1]. The intensity of the symptoms may vary depending on the quantity and swiftness of hemorrhage within the biliary tract, from silent, mild anemia to massive GI bleeding. Our patient presented with signs of hematemesis and hematochezia.

Hepatic artery pseudoaneurysm (HAP) can occur due to the same mechanism as hemobilia. HAP may be caused by acute or chronic damage to the hepatic artery. HAP with hemobilia is an unusual complication of blunt and penetrating liver trauma, prevalent in about 1.2% of cases with both trauma while prevalent in blunt trauma only cases if 0.6% [7]. Hemobilia is often accompanied by the presence of HAP, and it seems to signal its severity. Thus, when patients present with symptoms of hemobilia, HAP should be detected by imaging, and treated by embolization or surgery to rule out the cause of persistent GI bleeding and its fatal complications such as rupture. The majority of HAP are intrahepatic and occur in the right hepatic artery corresponding to the anterior segment of the right lobe; this is the most common site in blunt trauma of liver.

Ultrasound is a convenient and noninvasive method for monitoring injuries in trauma. It can reveal blood clots or echogenic intraluminal material in the gallbladder or biliary tree and may find mild biliary intra-extrahepatic dilation. Moreover, color Doppler can be useful to demonstrate the flow of an aneurysm [3, 8], occasionally showing a swirling blood flow within the pseudoaneurysm, described as the yin-yang sign [9].

Contrast-enhanced computed tomography (CECT) is routinely indicated for all cases of hemobilia to detect complications of liver injury such as an intrahepatic cavity and especially pseudoaneurysms [9]. CT may reveal spontaneous high-attenuation material such as blood clots within the gallbladder lumen and/or in the mild biliary duct dilatation. CT angiography reconstructed as three-dimensional (3D) volume-rendered and thick multiplanar reformation images from the arterial phase of CECT can be performed to evaluate the size, morphology, and location of pseudoaneurysm and its feeding artery to help plan for surgery or for embolization [10]. CECT may not find HAP if there is a very tiny pseudoaneurysm; this case had to be confirmed by hepatic angiography, and intervention can begin immediately if pseudoaneurysm appears [6, 11]. To avoid the risky complication of ruptured pseudoaneurysm as well as persistent hemobilia, early diagnosis and intervention is necessary.

Endoscopy and endoscopic retrograde cholangiopancreatography (ERCP) is recommended to rule out other more common causes of upper GI bleeding. It may help diagnose hemobilia, which is not detected on radiographic findings in blunt abdominal trauma [4]. However, because of the intermittent character of bleeding at the papilla of Vater, endoscopy may not find active bleeding in the majority of patients [3], so hemobilia should be suspected with the presence of blood clots in the GI tract.

In our case, despite closely monitoring, the diagnosis of hemobilia was not initially made until CT was done. Color Doppler should have been ordered in this case to confirm a false intrahepatic aneurysm. The patient underwent hepatic angiogram timely to finalize the diagnosis and complete the treatment.

Initial management of a patient with GI bleeding due to hemobilia can be conservatively achieved by fluid resuscitation, blood transfusion, and bed rest [12-13]. Although hemobilia may occasionally resolve spontaneously, most cases may require intervention. However, if pseudoaneurysms are not treated, there is potential for delayed rupture and acute life-threatening hemorrhage [9].

Surgical intervention, including partial hepatectomy, hepatic artery ligation, or pseudoaneurysm excision, is rarely necessary and usually reserved for failed endoscopic, endovascular, and/or percutaneous therapies. At present, this is rarely a therapeutic option. In some reports, blunt trauma patients developed hemobilia after their initial surgery and were evaluated with angiography, but embolization of one was not attempted, and for the other, not available; this instead required a laparotomy to control the bleeding [5, 10]. Transarterial angiography allows determination of the precise site of vascular injury, causing hemodynamically unstable hemobilia, and in most of these review cases, it becomes the modality of choice for both diagnosis and treatment. Endoscopic therapy has been considered as supportive treatment to existing methods [14-15].

Material embolizations include coils, glue such as histoacryl (NBCA), onyx, gel foam, polyvinyl alcohol (PVA) particles, but the most commonly used are coils and histoacryl. Coils are often used for their safety. One case presented a recurrent hemobilia after embolization of HAP and successfully treated by a second embolization [15]. This may be related to intimal damage during the intervention and to the reconstitution of hepatic collaterals and reformation of the HAP.

The embolic material NBCA (histoacryl) is also frequently used in the occlusion of HAP. In the present case, we used a packing technique, which can deploy the histoacryl mixture into the HAP. In a few cases, an isolation technique can be performed when the microcatheter tip could not readily approach the pseudoaneurysm, which excludes both pseudoaneurysm, its feeder, and distal branches from the circulation [16]. Histoacryl is diluted by mixing with lipiodol to help prolong polymerization time and radio-opacity. This ratio of histoacryl and lipiodol is varied, from 1:1 to 1:4, depending on the packing techniques with a higher mixing ratio or isolation techniques with a lower mixing ratio. Generally, although glue embolization of HAP has difficulties related to vascular anatomy and interventional techniques, it is still considered a fast procedure, easily performed, a low-cost treatment, and provides a permanent occlusion with a high success rate with minimal complications [3, 16].

Hemocholecystitis secondary to hemobilia has been published by several authors [5, 13, 17]. The gallbladder may accumulate blood over time and create blood clots, especially in patients with Oddi sphincter spasms. Mobile clots in the lumen (usually in the neck) may cause progressive gallbladder distension, resulting in mucosal damage and ischemia of the gallbladder wall [13]. Cholecystectomy is recommended in these cases.

## Conclusions

Although rare, hemobilia should be looked for during the treatment of blunt trauma to liver. Once GI bleeding is suspected, CT angiography is necessary to evaluate the development of biliary complications such as pseudoaneurysm and needs to be confirmed by transarterial hepatic angiography and its embolization as soon as possible. Moreover, depending on the case, alternatives such as endoscopy/ERCP or surgery may be used for the diagnosis and management of associated injuries and related complications that may occur during the treatment.
